# Supplementary material for: Amnesia after Midazolam and Ketamine Sedation in Children: A Secondary Analysis of a Randomized Controlled Trial
Source: J Clin Med. 2021 Nov 20;10(22):5430. doi: 10.3390/jcm10225430 (PMC8625279; doi:10.3390/jcm10225430)
Supplement: Supplementary file 1 [file jcm-10-05430-s001.zip › jcm-1349163-supplementary.pdf]

Table S1 - Characteristics of the children included in the qualitative analysis.

Child ID	Sex	Age (months)	Sedatives	Behavior	Sedation level
C01	Female	61	Oral midazolam	Negative	Minimal
C02	Male	73	Intranasal midazolam and ketamine	Positive	Minimal
C03	Female	60	Intranasal midazolam and ketamine	Negative	Minimal
C04	Female	52	Oral midazolam and ketamine	Positive	Minimal
C05	Female	43	Oral midazolam	Positive	Minimal
C06	Male	50	Oral midazolam	Negative	Minimal
C07	Female	43	Oral midazolam	Positive	Moderate
C08	Female	69	Intranasal midazolam and ketamine	Positive	Moderate
C09	Female	45	Intranasal midazolam and ketamine	Positive	Moderate
C10	Male	50	Intranasal midazolam and ketamine	Positive	Moderate
C11	Male	76	Oral midazolam and ketamine	Positive	Minimal
C12	Female	62	Oral midazolam and ketamine	Negative	Moderate
C13	Male	45	Oral midazolam and ketamine	Positive	Moderate
C14	Female	58	Intranasal midazolam and ketamine	Positive	Moderate
C15	Male	60	Intranasal midazolam and ketamine	Negative	Moderate
C16	Male	41	Oral midazolam	Negative	Moderate
C17	Male	43	Oral midazolam	Negative	Minimal
C18	Female	60	Oral midazolam and ketamine	Positive	Moderate
C19	Male	49	Oral midazolam and ketamine	Positive	Moderate
C20	Female	53	Oral midazolam	Positive	Moderate
C21	Female	42	Oral midazolam	Positive	Minimal
C22	Female	76	Intranasal midazolam and ketamine	Negative	Moderate
C23	Female	47	Intranasal midazolam and ketamine	Positive	Moderate
C24	Female	70	Oral midazolam	Negative	Moderate
C25	Male	70	Oral midazolam	Positive	Moderate
C26	Female	43	Oral midazolam	Positive	Moderate
C27	Female	48	Oral midazolam	Negative	Moderate
C28	Female	53	Intranasal midazolam and ketamine	Negative	Moderate
